# Synthetic epigenetics—towards intelligent control of epigenetic states and cell identity

**DOI:** 10.1186/s13148-015-0044-x

**Published:** 2015-03-04

**Authors:** Tomasz P Jurkowski, Mirunalini Ravichandran, Peter Stepper

**Affiliations:** Laboratory of Molecular Epigenetics, Institute of Biochemistry, Faculty of Chemistry, University of Stuttgart, Pfaffenwaldring 55, D-70569 Stuttgart, Germany

**Keywords:** Synthetic epigenetics, Targeted epigenome modification, Zinc fingers, TALE, CRISPR, Cell fate, Epigenetics, Epigenetic editing

## Abstract

Epigenetics is currently one of the hottest topics in basic and biomedical research. However, to date, most of the studies have been descriptive in nature, designed to investigate static distribution of various epigenetic modifications in cells. Even though tremendous amount of information has been collected, we are still far from the complete understanding of epigenetic processes, their dynamics or even their direct effects on local chromatin and we still do not comprehend whether these epigenetic states are the cause or the consequence of the transcriptional profile of the cell. In this review, we try to define the concept of synthetic epigenetics and outline the available genome targeting technologies, which are used for locus-specific editing of epigenetic signals. We report early success stories and the lessons we have learned from them, and provide a guide for their application. Finally, we discuss existing limitations of the available technologies and indicate possible areas for further development.

## Review

### Epigenetic landscape and cell fate

The human body is built from more than 200 different cell types organized in various tissues. It is fascinating that although all these cells stem from a single cell (the zygote) and contain exactly the same DNA sequence (except for the antibody-forming B cells of the immune system [1]), they express different sets of genes and have diametrically different functions, phenotypes and behaviour in the body. To explain how these phenotypic differences arise during embryonic development, in 1957 in his book “The Strategy of the Genes”, Waddington defined the famous concept of the *epigenetic landscape*, in which cells can be imagined as marbles rolling down towards a hill’s bottom. The marbles (cells) compete for the grooves on the slope, which define their developmental trajectories, and come to rest at the base of the hill in defined positions. These defined positions demarcate eventual cell fates, meaning the tissue types which the cells adapt [2].

The human genome comprises approximately three billion base pairs, which represent a large repository of information. The fact that different cells contain basically the same DNA but show very distinct phenotypes indicates that regulated access to this information is key to understanding cell identity and, therefore, human development and health. The last 60 years of research in the field of epigenetics was focused mostly on elucidating the molecular mechanisms and the enzymatic machinery responsible for epigenetic control of gene expression, as well as the distribution of various epigenetic marks in different cell types from healthy and diseased tissues and organisms. Progress in the next-generation sequencing and proteomic approaches allowed systematic analysis and identification of novel epigenetic marks and their distribution across the genome and nucleus. Few large-scale collaborative projects, like ENCODE (http://www.genome.gov/encode/) [3,4], Roadmap Epigenomics Mapping consortium (http://www.roadmapepigenomics.org/) [5] and Blueprint (http://www.blueprint-epigenome.eu/) [6] provided a validated repository of epigenetic states of various tissues.

Despite the tremendous progress in understanding the epigenetic signalling pathways, as well as characterization of the epigenetic marks (DNA and histone modifications) and the enzymatic machinery that can write, read and remove these marks, many fundamental questions still remain unresolved, mainly due to technological limitations. For example, are various epigenetic signals the cause or the consequence of the cell transcriptional profile? What is the sequential order of epigenetic transitions between the repressed and activated states? Are epimutations drivers or merely by-products of a diseased state, and finally, what is the contribution of epigenetics to disease (cancer) development [7,8]?

In this review, we try to define the area of synthetic epigenetics and outline the available genome targeting technologies, which are used for targeted editing of epigenetic signals. We report early success stories and the lessons we have learned from them, as well as potential biomedical applications and existing limitations of the available technologies.

### Synthetic epigenetics

We define synthetic epigenetics as *the design and construction of novel specific artificial epigenetic pathways or the redesign of existing natural biological systems, in order to intentionally change epigenetic information of the cell at desired loci*. In this broad definition view, somatic cell nuclear transfer experiments (SCNT) [9,10], direct cell fate conversion (also known as transdifferentiation) [11,12], generation of induced pluripotent stem cells (iPS cells) [13,14] and targeted epigenome editing by programmable DNA binding domains fused to epigenetic modifiers (epigenetic editing) [15] all constitute synthetic epigenetic phenomena. Due to their random nature of introduced epigenome modification (in terms of locus or sequence specificity), we do not consider epigenetic drugs (like azacytidine or trichostatin A [16,17]) as synthetic epigenetics tools.

Importantly, there is a qualitative difference between the nuclear transfer and induced pluripotency experiments when compared to epigenetic editing. In the experiments with somatic cell nuclear transfer and during generation of induced pluripotent stem cells, genome-wide changes in the epigenetic state of the cells are triggered by a mixture of defined oocyte-specific factors or forced expression of a selection of transcription factors, respectively. This is achieved through the restoration of robust pluripotency promoting transcription factor networks with self-enforcing feedback mechanisms, which rewire the transcriptional profile of the cell [18,19], and the epigenome is progressively adjusted during this process [20-22]. Interestingly, overexpression of some epigenetic modifiers, like ten-eleven translocation (TET) enzymes, can catalyse and enhance the dedifferentiation process [23,24]. Therefore, in these experiments, the observed epigenetic changes are rather a necessary product than the initial trigger of the cell conversion [25], which allow efficient reaching and maintenance of the de-differentiated state. This strategy can be viewed as an indirect (or top-down) approach to change the epigenome by rewiring the transcriptional profile of the cell. Somatic cell nuclear transfer and generation of induced pluripotent stem cells have been extensively covered in numerous excellent reviews [26-30].

On the other hand, in a bottom-up approach, a local direct change to the epigenome can be introduced by targeting an effector domain to the desired locus. In this epigenetic editing [15] approach, programmable DNA binding domains (DBDs) target a selected epigenetic modifier to desired loci and ensure deposition of the corresponding epigenetic mark at nearby chromatin. Consequently, local changes to the epigenome can be observed, and transcriptional and epigenetic response to these changes can be studied in a defined context.

Existing technologies used to modulate transcription of desired genes, like RNAi [31], gene knock-out experiments or expression of recombinant proteins (for example, overexpression of cDNA constructs), as well as programmable activators and silencers [32-36] require constitutive expression of the constructs to maintain the effect [34] or introduce irreversible changes to the genome. In contrast, epigenetic editing offers the possibility that the epigenetic signal and the corresponding change in the gene’s expression status are heritably maintained by cellular machinery over multiple cell divisions even after the initial epigenetic editing construct is cleared from the cells [34]. Therefore, a transient introduction of the construct can lead to a persistent modulation of gene expression without any genomic damage being introduced, hence making epigenetic editing safer and more suitable for therapeutic use. In this article, we will focus on the currently blooming and exciting field of targeted epigenome modification.

The epigenetic editing tools are perfectly suited to treat and study the molecular mechanisms underlying epigenetic diseases like cancer, chronic diseases or imprinting defects. For example, DNA methylation has been already used to silence overexpressed oncogenes [37-39] and could be further used to repress hypoxia-inducible factors [40], possibly leading to cancer regression. On the other hand, silenced tumour suppressor genes [41] could be reactivated using targeted DNA demethylation. Likewise, imprinting defects (for example, Beckwith-Wiedemann syndrome) could be reverted or their effects could be weakened by specific alteration of the epigenetic state of the affected imprinting control regions [42-44]. Chronic diseases are very often correlated with abnormal epigenetic changes [45-48]. With epigenetic editing technology, one could attempt to reprogram these disease-promoting epigenetic states and therefore restore normal functioning of the cell. An interesting new approach would also be to specifically change the differentiation state of pluripotent or differentiated cells, by rewiring their epigenetic profile towards another cell type.

### Bottom-up synthetic epigenetics or epigenetic editing

**Figure 1:**
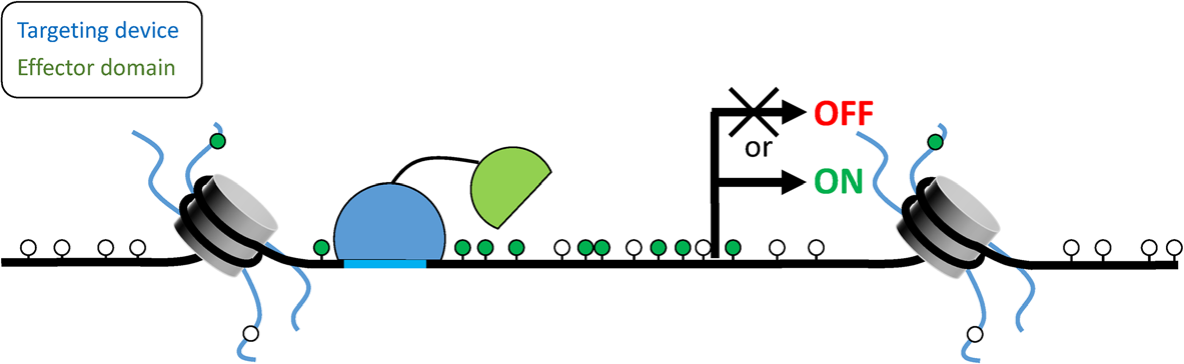
**The concept of epigenetic editing.** Targeting device, a sequence-specific DNA binding domain which can be redesigned to recognize desired sequences is fused to an effector domain, which can modify the epigenetic state of the targeted locus, leading to a persistent biological effect (gene activation or repression). *Green lollipops* represent introduced modification of either DNA bases or histone tails.

The concept of bottom-up synthetic epigenetics (epigenetic editing) relies on the combination of an artificial DNA binding domain, which can directly bind a unique sequence found within the desired locus, with an effector domain able to edit the epigenetic state of that locus (Figure 1). To date, various genome targeting domains and epigenetic modifiers have been used to direct activating or repressing marks to desired loci (reviewed in [15,49-51]). Discovery of novel programmable genome binders, like TALE and CRISPR/Cas9 systems, as well as progress in understanding of epigenetic enzymes has spurred new interest and brought excitement to the epigenetic editing field [52,53]. In 2014, *Nature Methods* has distinguished epigenome editing as a “Method to Watch” [54]. In the next paragraphs, we review the available technologies for epigenome targeting and lessons learned from the published success stories of epigenetic editing, and provide a guide for perspective researchers aiming to develop novel methods or apply existing ones for their own research.

### Genome targeting proteins

For most of the known DNA interacting proteins, no simple DNA recognition code exists that could connect individual amino acid residues with the corresponding DNA base [55]. For this reason, it was impossible for many years to redesign DNA interacting proteins for novel pre-defined specificities [56,57]. C2H2 zinc fingers were the first example of modular and predictable DNA recognition modules in which one zinc finger unit binds to three base pairs [58]. More recently, two more programmable genome binders were discovered: the TAL effector arrays [59] and CRISPR/Cas9 systems [60], which are discussed below.

### Zinc finger arrays

C2H2 zinc fingers were the first example of predictable DNA interaction domains amenable to rational protein design (reviewed in [58,61]), and until very recently, they were the domains of choice for sequence-specific genome targeting. Natural and engineered zinc finger arrays are composed of tandem repeated zinc finger modules. Each unit comprises around 30 amino acid residues that form a compact structure stabilized by zinc ions bound to two invariable cysteine and two histidine residues [62]. Separate zinc finger units were systematically modified and selected for recognition of various trinucleotides. Repositories of possible zinc fingers recognizing particular trinucleotide sequences are readily available [63,64]. Typical custom-made zinc finger arrays comprise between three and six individual zinc finger modules and can consequently bind target sites ranging from 9 to 18 base pairs in length. Arrays with six or longer zinc finger motifs are particularly interesting, as they can recognize target sites that are long enough to potentially address a unique sequence in the context of a mammalian genome.

Currently, two main methods are used to generate engineered zinc finger arrays: context-dependent modular assembly [65] and bacterial selection systems [66]. Context-dependent modular assembly (CoDA) relies on combining smaller zinc finger units of known specificity into larger arrays. Generation of custom zinc finger arrays with the use of a bacterial selection system has the potential to deliver highly efficient DNA binding modules. Because the zinc finger arrays, after their assembly from a large combinatorial library of shorter zinc finger modules, are tested right away in the cellular environment, the chances of obtaining well-functioning combinations of modules for a particular sequence are greatly increased. However, this process can be tedious and time consuming to have the arrays selected and validated [67]. On the other hand, CoDA can provide a large number of zinc finger arrays relatively quickly (within 1–2 weeks) [65], on the expense of the design’s success rate. Online tools for design of zinc finger arrays are available (http://zifit.partners.org/ZiFiT/ [68,69]).

### TAL effector arrays

Another class of customizable DNA binding domains, the transcription activating-like effectors (TALEs) are important virulence factors initially isolated from the bacterial plant pathogen *Xanthomonas* [70]. Members of the TALE family are composed of tandemly arranged and highly similar 34 amino acid repeats. Each repeat recognizes a single base pair, and the recognition specificity of TALEs correlates with the amino acid composition of the repeat variable di-residues (RVD) localized in positions 12 and 13 of each repeat [71]. Due to the modularity of TALEs and the restriction of base specificity to the RVDs, an elegant code governing the DNA recognition specificity was elucidated [59,72]. Because of the simplicity of the recognition code, the readout being restricted to a single base pair per repeat unit and the lack of neighbour effects, the custom TALE arrays can be efficiently generated by modular assembly [73]. However, both C2H2 zinc finger and TALE arrays need to be redesigned for each particular DNA sequence of interest, which is time and resource consuming.

### CRISPR/Cas9 system

The newest exciting addition to the genome targeting toolbox repository is the CRISPR/Cas9 system [74]. CRISPR (clustered regularly interspaced short palindromic repeats) functions as a prokaryotic adaptive immune system that confers resistance to exogenous genetic elements such as plasmids and phages [75]. In the natural system, short segments of foreign DNA (spacers) are incorporated into the genome between CRISPR repeats and serve as an adaptive memory of previous exposures [76]. CRISPR spacers are transcribed into non-coding precursor RNAs and further processed into mature CRISPR RNAs (crRNAs), which in turn guide CRISPR-associated (Cas) proteins to recognize and cleave invading genetic elements containing matching sequences [77]. Many CRISPR systems have been found in bacteria and archaea; they were categorized into three distinct types. Type II (referred here as CRISPR/Cas9) is the simplest, as it requires only one protein component for genome targeting, given that the appropriate guide RNA is provided. This system has been harnessed for genome engineering in a broad range of organisms [60,74]. The CRISPR/Cas9 protein requires a Cas9-specific protospacer-adjacent motif (PAM) sequence being present at the 3′-end of the targeted sequence for efficient binding and cleavage. CRISPR/Cas9 proteins recognize their targets based on Watson/Crick base pairing and rely on complementarity of the recognized DNA and the guide RNA sequences. Therefore, re-targeting of the guide RNA-Cas9 nuclease complex to a new locus only requires introduction of a new guide RNA sequence complementary to the new target sequence. In addition, orthologous Cas9 systems (isolated from diverse bacterial species) [78] fused to a selection of different epigenetic modifiers could be simultaneously used in a single experiment to target various epigenetic modifications to selected loci (same or different). These properties make the guide RNA/Cas9 system the most promising genome targeting approach available so far. For targeting epigenetic modification, a catalytically inactive Cas9 variant has to be used, which still can recognize and bind the target sequence, but cannot cleave it [78-80].

### Selection of the genome targeting proteins

Each of the available programmable genome targeting devices discussed above offers unique advantages and disadvantages (summarized in Table 1). When choosing the appropriate targeting domain for synthetic epigenetics application, few important properties should be taken into consideration: specificity of the target recognition, sensitivity to the state of DNA modification, ease of design and construct generation, as well as possibility for multiplexing (as discussed below).

**Table 1:** **Main characteristics of programmable genome targeting domains suitable for directing epigenetic modifiers**

	**Zinc fingers**	**TAL effectors**	**CRISPR/Cas9**
Origin	Eukaryotic species	Phytopathogenic bacteria	Bacterial and archaea species
Type of DNA recognition	Protein:DNA	Protein:DNA	RNP:DNA (Watson-Crick base pairing)
Function of the protein of origin	Transcription factors	Transcription factors	DNA nuclease (inactivated for use in epigenetic editing)
Sensitivity to DNA modification	Sensitive to DNA modification	Sensitive to DNA modification	Not sensitive to DNA modification state
Recognition sequence length	Potentially long, but not all sequences can be recognized, size restrictions apply	Potentially very long, constraints apply	17–20 bp, requires an adjacent PAM sequence
Specificity/off-target effects	Less specific than TALEs	Most specific	More relaxed sequence recognition than ZF and TALES
Size of protein	Variable—depends on length of recognized sequence, one protein unit (approximately 3 kDa) per 3 bp of recognition sequence	Variable—depends on length of recognized sequence. Typically 50-70 kDa	Holoenzyme (~160 kDa)
Immunogenicity	Similar to natural mammalian proteins, potentially low immunogenicity	Unknown, needs further investigation	Unnatural for mammals, potentially high immunogenicity, needs further studies
Multiplexing	Difficult and labour intensive	Difficult and labour intensive	Easy and possible

Unpredictable off-site targeting can result in the modification of epigenetic information at different loci than anticipated and thereby influence the obtained biological outcome, leading to false conclusions of the studies. Therefore, specificity of target recognition is of pivotal importance because strong binding to an off-target site, resulting from binding of the targeting device, could also lead to a stable, but unwanted epigenome modification around that region. On the other hand, the excess of epigenetic editors present in the cell is not expected to be deleterious. These surplus proteins could either be recruited by natural interaction partners to their native target sites and contribute to regular cellular processes or they could modify random sites on the genome, causing only minimal fluctuations of epigenetic signals that would be efficiently counteracted.

The specificity of zinc finger arrays will likely differ for each particular design [81]. TALE arrays are simpler in their DNA recognition code and assembly; therefore, the possible off-target sequences are more predictable. TALEs were also shown to be very selective in target binding [50,82]. It was demonstrated recently that the CRISPR/Cas9 system suffers from relaxed sequence specificity [83]. This is because not all the positions of the recognized sequence are read equally stringently [84], leading to frequent off-target binding [33,85]. However, successful attempts to improve the specificity have been reported as well [84,86]. Additionally, the requirement for the PAM motif limits the genomic sequences that can be targeted. Nevertheless, the recent solution of the crystal structure of the Cas9 protein bound to guide RNA and substrate DNA showing the mode of interaction between the PAM site and the Cas9 protein will hopefully facilitate directed evolution studies to re-engineer the PAM sequence requirement or even to completely remove the PAM dependency [87,88].

Both zinc fingers and TALEs read the sequence in the major groove of DNA [89,90]. Importantly, the known mammalian DNA modifications, 5-methylcytosine (5mC) and its oxidation products are also presented in the major groove of DNA and therefore can influence the binding of these domains to DNA [91,92]. There are examples of both natural and synthetic zinc finger proteins that recognize 5-methylated cytosine embedded in a specific DNA context [93]. Interestingly, Isalan and Choo have *in vitro* evolved zinc finger Zif268 protein to recognize and specifically bind HhaI (GCGC) and HaeIII (GGCC) methylated sites in a specific context, by using rounds of selection of phage displayed zinc finger randomized libraries that employed M.HhaI- and M.HaeIII-methylated DNA as baits [94]. Such methylation-specific zinc finger arrays could be employed for selectively targeting modified parts of the genome, for example, methylated gene promoters for targeted DNA demethylation and gene activation, provided that the exact methylation state of the promoter is known. In contrast, DNA sequence recognition of CRISPR/Cas9 relies on the Watson-Crick base pairing of the RNA guide and DNA sequence; therefore, it is not affected by DNA modifications found in mammalian genomes [95]. The modification state sensitivity of the zinc finger and TALE arrays should be taken into consideration while designing experiments, especially when the methylation status of the targeted region is not known. Additionally, certain TALE repeats have been reported to be insensitive to 5mC modification and therefore could be employed to overcome this limitation [91].

Overall, the CRISPR/Cas9 systems offer substantial advantages over zinc finger and TALE arrays, like simplicity of target design, possibility for multiplexing (targeting two or more sites at the same time) and independence of DNA modifications. Moreover, orthologous CRISPR/Cas9 proteins isolated from various bacteria could be employed for simultaneous targeting of different functionalities to the same or different loci [78]. However, relatively relaxed target specificity and potentially adverse immunogenicity might hamper their use in the clinic. In addition, the CRISPR/Cas9 system has not been employed for epigenetic editing yet (what is likely to be shown in the nearest future). On the other hand, zinc fingers and TALEs can offer superior target specificity and could be better tolerated by the immune system of a potential patient [96].

### Epigenetic modification domains (effector domains)

Selection of the appropriate effector domain is based on the planned application and intended effect on transcription and epigenetic state. Nature provides numerous possible modification domains, which can be employed for the particular function. Various effector modifiers have been already fused to zinc fingers, TALE arrays and other DNA binding domains to target their activities to endogenous or genome integrated targets, reporter plasmids or viral DNA [15,97]. Examples include DNA methyltransferases (bacterial M.SssI targeted *in vitro* to synthetic DNA [98], M.HpaII targeted to reporter plasmids and integrated viral DNA [99,100] and eukaryotic Dnmt3a catalytic domain and full-length proteins, Dnmt3a-Dnmt3L single-chain constructs targeted to endogenous loci [38,101]), ten-eleven translocation DNA demethylases (targeted to endogenous loci [102,103]), thymine DNA glycosylase (targeted to endogenous locus [104]), histone methyltransferases (G9a—targeted to integrated Gal4 binding site [105], G9a [106] and Suv39H1 [106,107] targeted to endogenous locus, Ezh2 targeted to reporter construct [108]) and histone demethylase (Lsd1 targeted to endogenous locus [109]), as well as histone deacetylases (targeted to reporter plasmid [110]), which could either activate or repress targeted genes. Interestingly, a light-controlled TALE system was used by the Feng Zhang and George Church laboratories to target histone modifiers and VP64 transcription activating domain. Even though the observed effects regarding histone modifications are modest (oscillating between 1.5-fold and 3-fold), this method in principle allows introduction of the mark not only at the desired locus, but also at a desired time [111].

Many (if not most) of the targeted epigenome modifications studies were focused on manipulating the DNA methylation state (either to specifically methylate or demethylate gene promoters), in order to repress active oncogenes or activate silenced tumour suppressor genes, respectively. This might be explained by the fact that in contrast to the not yet fully understood mechanism of maintenance of histone modifications during mitotic division [112], the mechanisms of setting up and inheritance of DNA methylation have been thoroughly studied (reviewed in [113]).

Methylation of gene promoters around transcription start sites and first exons is strongly correlated with gene repression [113,114]. Once DNA methylation is established, it is inherited after semiconservative DNA replication by the action of hemi-methylation-specific DNA methyltransferase Dnmt1 [115,116]. Therefore, targeted DNA methylation can provide a unique opportunity to heritably switch off gene expression (loss of function) [37-39]. It could be used for example to silence the overexpressed oncogenes by DNA methylation in cancer cells. On the other hand, targeted DNA demethylation, as shown in recent publications [102-104], offers an interesting way to activate the desired gene expression from its native locus (gain of function). Straightforward applications of these approaches for clinical studies could be, for example, to demethylate the promoters and thereby activate the expression of tumour suppressor genes, which are commonly silenced in cancer cells [117-120]. Moreover, new functions to differentiated cells could be conveyed by activating genes, which are normally not expressed in that cell type.

No matter which effector domain is selected for targeting, the applicability of that domain should include the evaluation of the extent and stability of the introduced modification and its biological effect, as discussed in the next sections.

### Stability of introduced epigenetic modifications

Despite the dynamic nature of epigenetic information, the overall cell state and global epigenetic states are heritable and maintained remarkably stably during multiple mitotic divisions. This is mainly due to the cooperation and redundancy of the multiple epigenetic signals (DNA and histone modifications), as well as to the transcriptional activity of each particular gene [121], which can reinforce the preservation of the current state. Thus, in contrast to the transient overexpression of transcription factors, modification of the epigenetic signal at selected promoters, through depositing of either activating or inactivating epigenetic marks, promises heritable maintenance of the induced state throughout multiple cell divisions. It is poorly investigated, however, if this assumption indeed holds true. Until now, most publications addressing directed epigenome modification did not investigate the long-term stability of the introduced mark, with few noble exceptions. Heritable DNA methylation and gene repression were observed after targeting zinc finger fused with the M.HpaII F35H mutant to the genomically integrated minimal thymidine kinase promoter governing expression of the CAT reporter gene construct. The introduced DNA methylation and the repressive effect were observed even 17 days post transfection of the zinc finger fusion constructs, when the expression of the ZF-HpaII F35H construct was no longer detectable neither at mRNA nor at protein levels [100]. However, in this report, an artificially introduced genomic locus was targeted; this study might therefore not represent a proof of concept for the stability of targeted DNA methylation at a natural genomic locus. Repression of a native tumour suppressor gene MASPIN locus was observed after its targeting by a zinc finger fused to Dnmt3a catalytic domain (CD) or KRAB transcription repression domain. Interestingly, after clearance of the construct from the cells, both the stable DNA methylation (up to 50 days post transfection) and the gene repression were maintained only in the case of ZF-Dnmt3a CD, but were lost in the case of ZF-KRAB transcription repression domain [34]. Even though the mechanisms of histone modification maintenance have not been completely understood at a molecular level, histone marks also seem to be stably maintained in the cells. In support of this, H3K27 methylation introduced by targeting Ezh2 next to the Gal4 binding site was maintained 4 days post clearance of the targeting construct [108]. Spreading and long-term stability (over multiple cell divisions) of H3K9me3 triggered by recruitment of HP1α to Oct4 promoter was also observed [122].

These studies illustrate that targeted epigenome modification can indeed withstand numerous cell divisions and is superior over effects introduced with transient targeting of repressors. Nonetheless, the introduced epigenetic modification might not be stable in all genomic contexts and will depend on the pre-existence of other activating or repressive epigenetic marks, the location of the locus in the euchromatic or heterochromatic region, as well as on the extent of the introduced modification and the nature of the mark itself. It is likely that small and local changes to the epigenome may not be efficiently maintained, and therefore, the locus would return to the initial state before perturbation happened [114,115]. Therefore, in order to achieve a stable effect, it might be beneficial to modify as big part of the region of interest as possible, either by employing a spreading mechanism or by targeting the fusion construct to multiple places within the same locus to achieve a cumulative effect [102]. In addition, simultaneous targeting of multiple epigenetic modifiers to the same locus, which reinforce the same effect, might strengthen the stability of the enforced new state.

### Spreading of the mark across the genome

**Figure 2:**
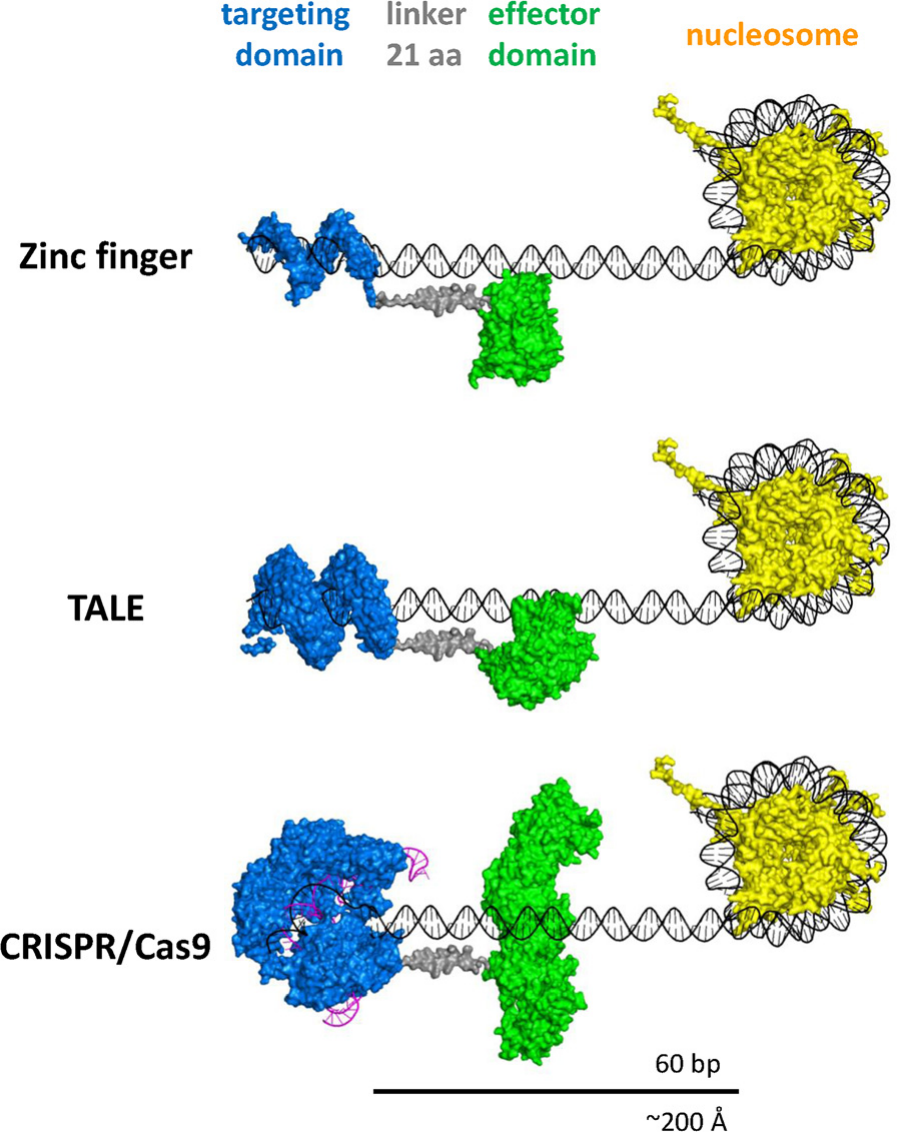
**Structural models of possible epigenetic editing devices.** The structural models of the proteins were taken from PDB repository (zinc finger [PDB:1P47], TALE [PDB:2YPF], CRISPR/Cas9 [PDB:4OO8], M.HhaI [PDB:5MHT], Dnmt3a/3L [PDB:2QRV], TET2 [PDB:4NM6], nucleosome [PDB:1AOI], a 21 amino acid linker was generated in PyMol, and 60 bp DNA sequence was generated with the make-na server (http://structure.usc.edu/make-na/server.html)). The models are drawn to scale and should provide an idea of the architecture of the synthetic constructs used for epigenetic editing. Modelling was done manually in PyMol. Zinc finger, DNA-bound zinc finger array fused to M.HhaI; TALE, synthetic TALE array fused to the catalytic domain of human TET2; CRISPR/Cas9, Cas9 protein fused to a Dnmt3a/Dnmt3L hetero-tetramer. The distances in base pairs and angstroms are indicated.

Since the epigenetic modifier is tethered to the DNA binding domain, which binds tightly to its recognition sequence, the length of the region which can be directly modified is limited. The extent of the linker between the targeting device and the epigenetic modifier is the main determinant of the possible reach distance. In the majority of the studies, the most efficient introduction of modification was observed in the nearest vicinity of the binding site of the targeting domain (10–40 bp [98,102,123]), which is in line with the typical distance that the linker region between the DBD and effector domain can provide. This is illustrated in Figure 2, which shows models of possible epigenetic targeting constructs with the DNA sequence and nucleosomes drawn to scale. Of course, if extensive DNA bending and looping are considered, the modification could reach further.

Due to mechanical constraints of the targeted epigenetic editing machinery, it is unlikely that a single and even very stable binding event would lead to a widespread modification at larger distances. Interestingly, histone modification [106] and DNA methylation spreading was observed beyond the expected distance that could be reached when considering the provided domain linker. We and the others observed the deposition of DNA methylation marks up to 300 bp and more from the targeted site when using the catalytic domain of Dnmt3a (or the Dnmt3a-Dnmt3L single-chain construct) to human EpCAM and VEGFA promoters [37,38]. Theoretically, this observation can be explained by extensive looping of the DNA in this region, which in turn would allow the tethered DNA methyltransferase to reach more distant regions of the DNA sequence. In a more interesting hypothesis, the spreading might be explained by a polymerization of Dnmt3a on the EpCAM and VEGFA promoters. Indeed, it has been shown that Dnmt3a cooperatively polymerases along the DNA molecule [124-126] and that its methylation activity is stimulated by the filament formation [127]. In this model, the targeted molecule of Dnmt3a would recruit additional molecules of the enzyme (maybe even the endogenous protein) to the modified region, leading to an efficient methylation of a larger genomic region adjacent to the targeted sequence (nucleation point). Whether the experimentally observed broad modification of these regions is due to DNA looping and nucleosome wrapping or the proposed spreading mechanism needs to be further investigated.

Whereas most of the studies were designed to achieve widespread DNA methylation of the targeted locus, Chaikind and colleagues developed an opposite strategy to selectively methylate a single CpG site in the genome using a split enzyme approach [128,129]. Two inactive parts of a DNA methyltransferase (M.SssI, M.HhaI) are separately directed to sites flanking the selected CpG, where they assemble to an active enzyme and methylate that target site, thus allowing to study the epigenetic consequences of a single methylation event. Additionally, the split enzyme approach would limit the extent of off-target effects, because the functional enzyme is only reconstituted at the targeted locus.

### Delivery of programmable epigenetic editors for epigenetic therapy

The efficiency of introducing epigenetic change and therefore possible biomedical application depend in big part on the vehicle used for delivery of the constructs. So far, only cultured cells and not whole organisms were used for epigenetic editing applications. However, similar delivery methods as used for genome engineering can be applied for this purpose as well. Besides the traditional approaches, like transfections of transient expression plasmids, transduction of adeno-associated viral (AAV) [130], lenti-, retro- or adeno-viral vectors, new ways to deliver the gene or protein cargo to the cells have been discovered. One intrinsic problem with the aforementioned viral delivery systems is their limited insert size capacity (in particular AAV and lenti-viruses), which can become a restricting factor because the epigenome editing constructs tend to be very large (in particular Cas9 fusions) and targeting of multiple loci at once may become difficult. To overcome these constraints, new delivery strategies were developed. An interesting approach is the hydrodynamic injection method, in which the plasmids encoding the targeting constructs are injected directly into the bloodstream of an animal. Subsequently, the cells can internalize the DNA and express the protein, which can in turn exert the desired effect in the cells [131]. Another interesting novel approach is to deliver purified proteins to the cells by attaching cell-penetrating peptides to the purified protein constructs or RNA:protein complexes (in the case of CRISPR/Cas9), therefore allowing spontaneous uptake by the cells [132-134]. Likewise, zinc finger arrays were shown to be intrinsically cell permeable and therefore could be easily delivered [135]. However, not all the proteins are intrinsically cell permeable. To overcome this limitation, in the most recent report, the authors have tethered TALE-VP64 proteins with negatively supercharged domains (containing large amounts of acidic residues) to deliver the proteins using poly-cationic transfection reagents. Interestingly, Cas9 when complexed with guide RNA could be delivered without this additional domain [136].

## Conclusions

From the time when the field of epigenetics was defined by Waddington until nowadays, we have learned a lot about epigenetic regulation of gene expression and maintenance of cellular identity. Substantial progress was achieved in identification and understanding the role of various epigenetic marks, their distribution in healthy and diseased tissues and the enzymatic machinery responsible for depositing, reading and removing these marks. This progress was accompanied or rather preceded by technological development, like chromatin immunoprecipitation (ChIP), bisulfite sequencing, high-throughput proteomic and whole genome approaches. The bottom-up synthetic epigenetics (targeted epigenome editing), although still in its infancy, constitutes an area of extensive research. New technological developments will likely increase the specificity of targeting devices and the efficiency of effector domains in setting the desired epigenetic marks and will supply engineered systems for spreading the modification across the whole locus, providing efficient and reliable tools for stable modification of the epigenome. The most exciting progress is expected from the CRISPR/Cas9 system, as it allows the biggest flexibility and ease of design of new targets and the possibility to construct target libraries [137,138], which will allow unprecedented control of the epigenetic states at desired loci.

Synthetic epigenetics has the potential to address so far unapproachable areas of basic and clinical research. It provides tools and methods, which allow dissection of the epigenetic signalling cascades and to identify the driver and passenger modifications. It can widen our understanding of epigenetic dynamics and the basis of signal inheritance. Top-down synthetic epigenetic approaches have already made a big contribution in generating disease model cell lines. Epigenetic editing will further foster biomedical research by addressing the epigenetic contribution to complex and simple diseases, through discovery and validation of disease-promoting epimutations and provide means for reverting them. It also offers tools to probe factors responsible for cell identity and allows intelligent control of the cell fate.

## Abbreviations

5mC: 5-Methylcytosine
Cas9: CRISPR-associated protein 9
ChIP: Chromatin immunoprecipitation
CRISPR: Clustered regularly interspaced short palindromic repeats
DBDs: DNA binding domains
Dnmt: DNA methyltransferase
EpCAM: Epithelial cellular adhesion molecule
Ezh2: Enhancer of zeste 2
iPS cells: Induced pluripotent stem cells
KRAB domain: Krüppel-associated box domain
PAM: Protospacer adjacent motif
RNP: Ribonucleoprotein
RVD: Repeat variable di-residues
SCNT: Somatic cell nuclear transfer
TALE: Transcription activating-like effector
TET: Ten-eleven translocation
VEGFA: Vascular endothelial growth factor A
ZF: Zinc finger
